# An investigation of minimisation criteria

**DOI:** 10.1186/1471-2288-6-11

**Published:** 2006-03-15

**Authors:** Angie Wade, Huiqi Pan, Simon Eaton, Agostino Pierro, Evelyn Ong

**Affiliations:** 1 Centre for Paediatric Epidemiology and Biostatistics, Institute of Child Health, University College, 30 Guilford Street, London, UK; 2 Department of Paediatric Surgery, Institute of Child Health and Great Ormond Street Hospital, 30 Guilford Street, London, UK

## Abstract

**Background:**

Minimisation can be used within treatment trials to ensure that prognostic factors are evenly distributed between treatment groups. The technique is relatively straightforward to apply but does require running tallies of patient recruitments to be made and some simple calculations to be performed prior to each allocation. As computing facilities have become more widely available, minimisation has become a more feasible option for many. Although the technique has increased in popularity, the mode of application is often poorly reported and the choice of input parameters not justified in any logical way.

**Methods:**

We developed an automated package for patient allocation which incorporated a simulation arm. We here demonstrate how simulation of data can help to determine the input parameters to be used in a subsequent application of minimisation.

**Results:**

Several scenarios were simulated. Within the selected scenarios, increasing the number of factors did not substantially adversely affect the extent to which the treatment groups were balanced with respect to the prognostic factors. Weighting of the factors tended to improve the balance when factors had many categories with only a slight negative effect on the factors with fewer categories. When interactions between factors were included as minimisation factors, there was no major reduction in the balance overall.

**Conclusion:**

With the advent of widely available computing facilities, researchers can be better equipped to implement minimisation as a means of patient allocation. Simulations prior to study commencement can assist in the choice of minimisation parameters and can be used to justify those selections.

## Background

Most medical researchers are aware that it is necessary to perform a randomised controlled trial to effectively establish the usefulness of a new treatment. The aim is that treatments are compared on similar groups of patients. Completely random allocation of patients to treatments does not, however, ensure that the patient groups are similar with respect to prognostic factors. For example, purely by chance one of the treatment groups may have been allocated older or more severely ill patients. If such an imbalance in prognostic factors has occurred then it may be difficult to attribute any differences to treatment, the analyses will require adjustment and the study will have less power.

Minimisation [1-3] is a dynamic allocation procedure that ensures treatment groups are similar with respect to a series of pre-specified prognostic factors. As patients are recruited to the trial they are allocated to the treatment group that will 'minimise' the differences in the distribution of those factors between the groups. One pitfall of this process is that the allocation is not random and hence could be predicted. This problem is addressed by randomising each patient but weighting the randomisation towards the minimisation favoured treatment group for that individual. By introducing weighted randomisation, the individual is more likely to be allocated to receive the preferred treatment, but is not guaranteed to do so. There is a trade-off between the size of the weighting used and the ability of the researcher to predict the next allocation.

To apply minimisation requires only simple algebra but this may be problematic for the clinician with limited time and resources. The advent of greater access to computing technology has led to an increase in the usage of minimisation, which has previously been implemented relatively rarely. Published trials increasingly cite the use of minimisation for patient allocation. For example, Falk et al [4] minimized patients to receive immediate or delayed radiotherapy with groups balanced according to clinician, histology, presence of metastases and WHO performance status. Pal et al [5] used minimisation to ensure even distribution within age groups (2–12 or 13–18 years) and whether or not there was cerebral impairment when conducting a trial of phenobarbital versus phenytoin for seizure control amongst epileptic children in rural India. Minimisation can similarly be used for allocation within cluster randomised controlled trials when confounding factors are applicable at the cluster level. For example, Hilton et al [6] performed a cluster randomised trial of intervention to lessen individuals' cardiovascular risk with general practices allocated to intervention or control using minimisation for Jarman score, ratio of patient to practice nurse hours per week and fundholding status.

The benefits of minimisation have been debated recently [7,8] and a recent review of the usage of minimisation recommended 'its wider adoption in the conduct of randomized controlled trials' [9].

The technique has not been without its critics [10] as well as advocates, but it is acknowledged to be relatively simple to implement and perform comparably to more complex models where prognostic factors are non-numeric in basis [11]. A common criticism is that minimisation concentrates only on marginal distributions of prognostic factors and may not ensure that the interactions are similar between groups. For example, although there may be similar numbers of males, females, disease state positive and negative in the treatment groups post allocation, all of the positive males may receive one treatment and all of the positive females the other. If there is an interaction between disease state and gender on outcome, then this difference between the treatment arms may be problematic. However, the likelihood of imbalance is easily countered via a 4-category minimisation variable: male/positive, male/negative, female/positive, female/negative.

The usefulness of minimisation as an allocation procedure within randomised controlled trials is therefore established and will continue to be recognised and utilised. However, it has been commonplace for published studies that have used minimisation to give little or no information as to how the process has been implemented. Often they cite the minimisation variables but do not state what metric has been used to determine the preferred allocation group, whether and what randomisation weightings have been used for the allocations, whether and how the factors have been weighted, whether interactions have been accounted for or, where applicable, how cut-points for continuous prognostic factors were selected. The researcher who wishes to embark on a trial using minimisation as the means of patient allocation often has many questions to ask. Several parameters need to be selected for each trial to which minimisation is to be applied. These parameters and, from our experience within the statistical consultancy, the most common associated questions related to the choice of each, are given below:

1. Number of factors

• How many factors can be balanced simultaneously?

• How does the balance for an individual factor change as more factors are incorporated?

• In particular, what is the effect on other factors of adding a single, many-categoried factor such as centre?

2. Number of categories for each factor

• How does choice of number of categories (for continuous prognostic factors) affect the allocation process?

3. Whether and how to weight the factors

• How should the factors be weighted?

4. The randomisation weighting to use

• How much should the randomisation be weighted in favour of the preferred group?

They also want to know

• With a chosen randomisation weighting and given number of minimisation factors, how big a discrepancy can be expected for a specified sample size?

and

• How does inclusion of interactions between prognostic factors change all this?

There is minimal information available in the published literature to inform researchers when addressing the above questions.

We have developed a simple computer package to perform minimisation allocations subject to selected values of these 4 input parameters. We have also incorporated a simulation element that allows the researcher to investigate the size of discrepancies in allocation of prognostic factors between treatment groups subject to variation in the input parameters.

We here present the results of some simulations for a hypothetical proposed trial and show how this process can assist the researcher in deciding on the parameter values to be used in their trial. Subject to our chosen criteria for model comparison, we examine the extent to which varying the minimisation parameters may influence the equalisation of prognostic factors between treatment groups.

## Methods

For each new patient to be allocated, the process of minimisation considers the imbalance in selected prognostic factors and weights the randomisation of the next patient, according to his or her characteristics, in favour of the treatment that will make the treatment groups most similar with respect to the prognostic factors. To formalize this process, assume a trial where patients are allocated to one of two treatments, T1 and T2. Suppose there are M prognostic factors and c_*j *_is the number of categories for the *j*^th ^prognostic factor (*j *= 1,...,M). Let a_nj _be the value that the n^th ^patient takes for the *j*^th ^prognostic factor (a_nj_∈ {1,2,...,c_j_}) and let *d*_j _be a measure of the difference between the numbers of patients allocated to each of the two treatments who are in category a_nj _after allocation of the (n-1)^st ^patient. Assume *d*_j _positive if more patients in category a_nj _are currently receiving T2, negative if more are receiving T1 and zero if both treatments currently contain equal numbers of patients at this level for the j^th ^confounder. Therefore D_n-1 _= {*d*_1_, *d*_2_, ..., *d*_*M*_} is the vector of differences between the treatment groups with respect to the M prognostic factors at the levels seen in the n^th ^patient prior to allocation of that patient. Using minimisation, according to the size and direction of D_n-1_, randomisation of the n^th ^patient will be weighted towards the treatment group that will make D_n _numerically smaller according to some chosen metric ie. the differences between the treatment groups will be minimised with respect to the prognostic factors.

For example, consider a study where there are M = 4 minimisation criteria: gender, age (under/over 18), residency status of patient (in/out) and severity of disease (mild/moderate/severe). Suppose that 34 patients have been recruited and allocated to one of 2 treatment arms (17 per arm) and that they are distributed amongst the minimisation criteria categories as follows:

**Table 1:** 

	Treatment arm:	
	T1	T2
Gender:		
Male	8	9
Female	9	8
Age:		
Under 18	14	12
Over 18	3	5
Residency status:		
In patient	7	7
Out patient	10	10
Severity of disease:		
Mild	4	3
Moderate	12	11
Severe	1	3

Suppose that the 35^th ^(n = 35) patient to be allocated (n = 35) is an adult male in-patient with mild disease. Prior to his allocation the numbers of patients falling into these categories is 8+3+7+4 = 22 in treatment arm T1 and 9+5+7+3 = 24 in treatment arm T2. Hence D_34 _= {8-9, 3-5, 7-7, 4-3} = {-1, -2, 0, 1} is the vector of differences between the treatment groups with respect to the 4 prognostic factors at the levels seen in the 35^th ^patient prior to allocation of that patient. Since there are fewer similar patients in T1, randomisation of the 35^th ^patient should be weighted towards this group.

### Choice of metric to minimise D_n_

The simplest algorithm for allocation of the n^th ^patient is to weight the randomisation in favour of T1 if ∑j=1Mdj>0
 MathType@MTEF@5@5@+=feaafiart1ev1aaatCvAUfKttLearuWrP9MDH5MBPbIqV92AaeXatLxBI9gBaebbnrfifHhDYfgasaacH8akY=wiFfYdH8Gipec8Eeeu0xXdbba9frFj0=OqFfea0dXdd9vqai=hGuQ8kuc9pgc9s8qqaq=dirpe0xb9q8qiLsFr0=vr0=vr0dc8meaabaqaciaacaGaaeqabaqabeGadaaakeaadaaeWbqaaiabdsgaKnaaBaaaleaacqWGQbGAaeqaaOGaeyOpa4JaeGimaadaleaacqWGQbGAcqGH9aqpcqaIXaqmaeaacqWGnbqta0GaeyyeIuoaaaa@383E@, in favour of T2 if ∑j=1Mdj>0
 MathType@MTEF@5@5@+=feaafiart1ev1aaatCvAUfKttLearuWrP9MDH5MBPbIqV92AaeXatLxBI9gBaebbnrfifHhDYfgasaacH8akY=wiFfYdH8Gipec8Eeeu0xXdbba9frFj0=OqFfea0dXdd9vqai=hGuQ8kuc9pgc9s8qqaq=dirpe0xb9q8qiLsFr0=vr0=vr0dc8meaabaqaciaacaGaaeqabaqabeGadaaakeaadaaeWbqaaiabdsgaKnaaBaaaleaacqWGQbGAaeqaaOGaeyOpa4JaeGimaadaleaacqWGQbGAcqGH9aqpcqaIXaqmaeaacqWGnbqta0GaeyyeIuoaaaa@383E@ and use simple randomisation (*p *= 1/2, where *p *is the probability with which the patient is allocated to the preferred treatment) if ∑j=1Mdj>0
 MathType@MTEF@5@5@+=feaafiart1ev1aaatCvAUfKttLearuWrP9MDH5MBPbIqV92AaeXatLxBI9gBaebbnrfifHhDYfgasaacH8akY=wiFfYdH8Gipec8Eeeu0xXdbba9frFj0=OqFfea0dXdd9vqai=hGuQ8kuc9pgc9s8qqaq=dirpe0xb9q8qiLsFr0=vr0=vr0dc8meaabaqaciaacaGaaeqabaqabeGadaaakeaadaaeWbqaaiabdsgaKnaaBaaaleaacqWGQbGAaeqaaOGaeyOpa4JaeGimaadaleaacqWGQbGAcqGH9aqpcqaIXaqmaeaacqWGnbqta0GaeyyeIuoaaaa@383E@. This system of allocation assumes equal weighting for the M prognostic factors.

The prognostic factors can be given different weightings (w_j_) according to their relative importance and ∑j=1Mwjdj
 MathType@MTEF@5@5@+=feaafiart1ev1aaatCvAUfKttLearuWrP9MDH5MBPbIqV92AaeXatLxBI9gBaebbnrfifHhDYfgasaacH8akY=wiFfYdH8Gipec8Eeeu0xXdbba9frFj0=OqFfea0dXdd9vqai=hGuQ8kuc9pgc9s8qqaq=dirpe0xb9q8qiLsFr0=vr0=vr0dc8meaabaqaciaacaGaaeqabaqabeGadaaakeaadaaeWbqaaiabdEha3naaBaaaleaacqWGQbGAaeqaaOGaemizaq2aaSbaaSqaaiabdQgaQbqabaaabaGaemOAaOMaeyypa0JaeGymaedabaGaemyta0eaniabggHiLdaaaa@393D@ used to determine the allocation. There are a variety of ways that the w_j _may be chosen. One potential system that seems reasonable is to weight according to the number of categories. For example, if 100 patients are allocated to two treatment groups then we would expect 25 within each category of a binary variable for each treatment group, and 10 within each category of a 5-category variable for each treatment (assuming, without loss of generality, equal probability of the categories occurring within each variable). A maximum difference of the same absolute magnitude between the two treatment allocations for any of the variable categories would probably be more clinically relevant for the 5-category than for the binary variable:

**Table 2:** 

T1	T2	Absolute Difference	T1	T2	Absolute Difference
20	30	10	5	15	10
25	25	0	10	10	0
			10	10	0
			10	10	0
			10	10	0

The absolute (unweighted) differences are identical (= 10) but for the binary variable T2 contains 50% more patients within the first category, for the 5-category variable there are 200% more relative to T1. The deviations from expected are 20% (5/25) and 50% (5/10) respectively. Weighting the absolute differences (= 10) by the number of categories gives weighted differences of 20 (10 × 2) and 50 (= 10 × 5) for the 2 and 5 category variables respectively.

The extent to which randomisation is more likely to favour one treatment over the other depends on the size of the randomisation weighting used. With *p *= 1/2 (simple randomisation) there is no preference for either group. When *p *= 1, the patient automatically receives the preferred treatment, there is no random element and allocation is said to be deterministic i.e. the researcher could predict the group allocation of the next patient if they knew the previous allocations. Selecting a value 1/2 <*p *< 1 will bias allocation in favour of the preferred treatment whilst retaining an element of randomness so that patient allocation cannot be predicted. More extreme values of *p *(greater bias) will lead to better balancing of prognostic factors between treatment groups.

There must be a trade-off between introducing sufficient randomisation weighting (p large enough) and not allowing allocation of the next patient to be predicted (p not close to 1).

### Quantification of balance

The maximum absolute difference between the patients allocated to each of the categories within a given factor summarises how well that factor has been balanced by the minimisation process and is used as a summary measure of balance for that factor. In our simulations we summarise across factors with the same number of categories. For example, if we minimize according to 3 binary variables and one 15-category factor then there will be 2 summary measures of balance achieved for each simulated dataset: the maximum absolute differences allocated to (1) any of the binary variables i.e. the 6 categories which constitute these 3 factors and (2) any category within the 15 category factor.

As previously noted [11] it is the behaviour of an individual design that is of interest to the researcher rather than the average over many applications. Of interest is the value below which discrepancies are likely to occur for the majority of applications of minimisation with given criteria. An individual investigator is more likely to want to know the maximum discrepancy that s/he can reasonably expect with chosen allocation parameters rather than what the average will be for everyone using those parameters. For these reasons we prefer the 95^th ^centile of the simulated distributions as more realistic and relevant measures than the means or medians which have previously been used to describe the success of a selected allocation process. The error in estimating the 95^th ^centile is about 1 1/2 times the error when estimating the mean [12] but this difference becomes unimportant if a large number of simulations are taken.

The 95^th ^centiles of the distributions of simulated values are used to compare allocation schemes with varying input parameters (sample size, randomisation weighting, number and type of variables, weighting of variables).

### Simulation options

It was estimated that 200 simulations would allow quantification of the 95^th ^centile of the distribution to within ± 0.2 standard deviations of that distribution with 95% confidence. Increasing the number of simulations to 2500 or 5000 would increase this precision substantially to ± 0.06 and 0.04 respectively. A further doubling of the number of simulations (to 10000) would only further increase precision by less than 0.01 standard deviations. Hence it was decided that 5000 simulations would be adequate for a comparison of minimisation criteria. For each scenario 5000 simulations were performed using a Fortran program incorporating NAG subroutines for random selection of patient characteristics.

#### Measure of balance

The 95^th ^centiles of the distributions of maximum absolute differences for each factor type were recorded and classified according to the number of potential categories within which each individual could fall. These differences are expressed as the proportionate difference from that expected by multiplying by the number of categories for that factor and dividing by the total sample size.

#### Randomisation weighting

All simulations were repeated for *p *set at 1/2 (simple randomisation) and also for *p *taken to be 0.67, 0.75, 0.8, 0.83, 0.88, 0.91, 0.95, 0.97, 0.99 and 0.999, values which equate respectively to allocation to the preferred treatment being 2, 3, 4, 5, 7, 10, 20, 30, 100 and 1000 times as likely as to the alternative.

#### Weighting of variables

Simulations were performed with prognostic factors both unweighted (*w*_j _= 1; j = 1,...,M) and with weights equal to the number of categories of the prognostic factor.

To address the types of questions posed by potential researchers we here simulate several different scenarios:

Firstly, we investigate the effect on balance of increasing the number of factors. Models with 1, 2, 3, 4, 5, 10, 20 and 30 binary prognostic factors are compared. The sample size for each simulation was set at 500. Since all factors are binary, weighting will not change the results and these models are only presented unweighted.

Secondly, we chose a study with three binary, one 3-category and one 4-category (total 5) prognostic factors to simulate. This scenario was chosen as being typical of the problems we were encountering in the local consultancy and not dissimilar to published studies citing minimisation criteria which commonly have several binary criteria and one or more multiple category confounders [4,6]. We investigated the extent to which balance was a function of sample size by simulating the scenario with each simulation based on samples of 40 and of 500 patients. For sample size of 500, we also considered the balance achieved when all 2-way interactions were used as the minimisation criteria (total of 10 interactions between the 5 confounders). Finally, we generated simulations of allocations for a sample size of 500 based on 6 minimisation factors: the original 5 plus an additional 15- category confounder.

### Practicalities of application

The practicalities of application require some degree of automation. We have developed a package for clinical usage (SiMin) which both utilises simulations to facilitate the process of specification selection and provides a user-friendly front-end for the subsequent allocations within trial. The Fortran programs which generate the simulations are embedded in this package. The purpose of this paper is to illustrate the types of patterns that can easily be determined via simulation and to show how this might assist the researcher, leading to more informed parameter selection and enhanced reporting of the decision process.

## Results

All results were equivalent for randomisation weights of 100 and 1000. Hence, only the results for 100 are shown in the figures.

### Increasing the number of factors

**Figure 1:**
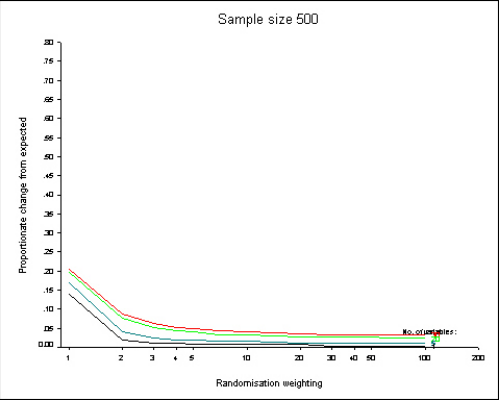
**Increasing the number of binary factors**. 95^th ^centiles of the distributions of proportionate changes from expected for randomisation weights from 1 to 100 obtained from 5000 simulations and a simulated sample size of 500. The number of minimisation variables is increased from 1 (black line) to 5 (dark green), 20 (lime green) and to 30 (red).

Figure 1 shows the results of the simulations as the number of binary factors is set at 1, 5, 20 and 30. The output for 2, 3, 4 and 10 binary factors are not shown on the figure. As expected, the proportionate change from expected falls as the randomisation weighting is increased. The largest fall occurs after the introduction of any randomisation weighting i.e. between values of 1 (simple randomisation) and 2. As the randomisation weighting is increased to 5 in favour of the preferred treatment there are further declines and less so between 5 and 30. After 30 the proportionate change appears to have reached an asymptote. The differences in proportionate changes with increasing number of factors are approximately constant with changing randomisation weights.

### Weighting the variables

**Figure 2:**
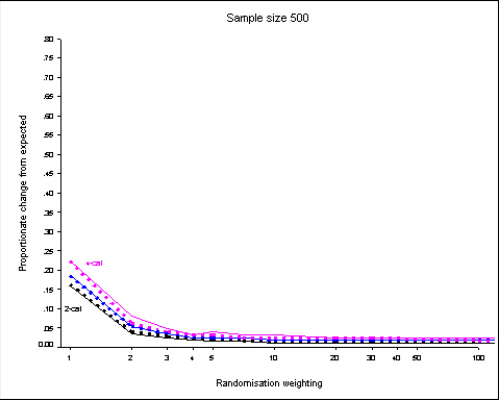
**Sample size 500, 5 prognostic factors (3 binary, 1 3-category, 1 4-category)**. 95^th ^centiles of the distributions of proportionate changes from expected for randomisation weights from 1 to 100 obtained from 5000 simulations. Dotted lines show results when prognostic factors are weighted according to the number of categories. Results for binary factors shown in black, 3-category in blue and 4-category in purple.

Figure 2 shows the 95^th ^centiles of the distributions of proportionate changes for the 5 prognostic factor (3 binary, 1 3-category and 1 4-category) scenario with a sample size of 500. The dotted lines show the results when the prognostic factors are weighted according to the number of categories.

Again, any degree of randomisation weighting is associated with the largest fall in change from expected. Proportionate differences increase with the number of categories. Weighting of the prognostic factors has the effect of increasing the discrepancies for the binary factors but reducing them for the factors with more categories.

### Changing the sample size

**Figure 3:**
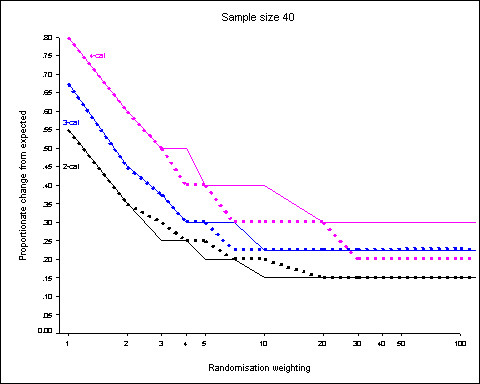
**Sample size 40, 5 prognostic factors (3 binary, 1 3-category, 1 4-category)**. 95^th ^centiles of the distributions of proportionate changes from expected for randomisation weights from 1 to 100 obtained from 5000 simulations. Dotted lines show results when prognostic factors are weighted according to the number of categories. Results for binary factors shown in black, 3-category in blue and 4-category in purple.

Figure 3 shows the 95^th ^centiles of the distributions when a sample of only 40 patients is allocated using 5 minimisation factors (3 binary, 1 3-category and 1 4-category). The proportionate differences are much larger with the smaller sample size (c.f. figure 2).

### Interactions

**Figure 4:**
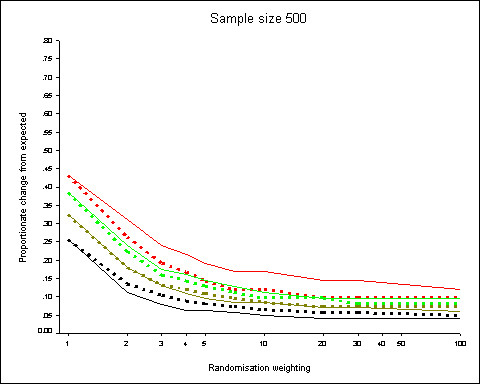
**Minimizing the difference of the interactions**. 95^th ^centiles of the distributions of proportionate changes from expected for randomisation weights from 1 to 100 obtained from 5000 simulations. All 10 2-way interactions from 5 prognostic factors (3 binary, 1 3-category, 1 4-category) used as minimisation criteria. Dotted lines show results when prognostic factors are weighted according to the number of categories. Results for the 3 2 × 2 interaction terms shown in black, for the 3 2 × 3 interactions in dark green, the 3 2 × 4 in lime green and the 1 3 × 4 interaction in red.

Figure 4 shows 95^th ^centiles of the distributions of proportionate changes when minimisation is used to allocate 500 patients to 2 groups with the aim of obtaining even distribution of all 2-way interactions of 5 confounders (3 binary, 1 3-category and 1 4-category). If the distributions sof the interactions are similar, then the marginal distributions of the factors will be also.

The differences are increased from the non-interaction model (Figure 2) approximately 2-fold for the lower randomisation weights (and when simple randomisation is used) up to approximately 5-fold for large randomisation weighting. Since the proportionate changes are smaller for larger randomisation weights, the absolute difference in the proportionate changes falls with increasing randomisation weights. It should be noted, however, that the interaction factors have more categories and the discrepancies are about the same in terms of the numbers of individuals allocated.

The effect of weighting of the prognostic factors is similar to previous models. When factors are weighted according to the number of categories this has the effect of reducing the proportionate discrepancies for the factors with more categories (3 × 4 and 2 × 4 interactions) whilst having the opposite effect for the factors with fewer categories (2 × 2 and 2 × 3 interactions).

### Adding a factor with many categories

**Figure 5:**
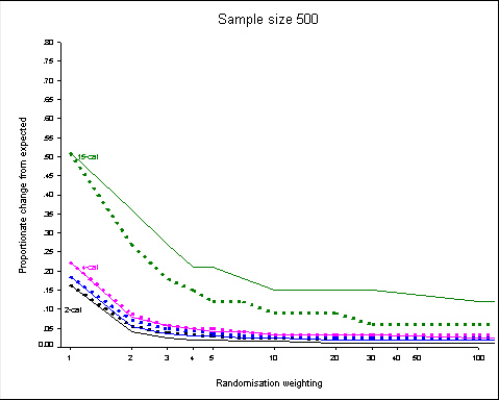
**Adding a 15-category factor**. 95^th ^centiles of the distributions of proportionate change from expected for randomisation weights from 1 to 100 obtained from 5000 simulations. Dotted lines show results when prognostic factors are weighted according to the number of categories. Results for binary factors shown in black, 3-category in blue, 4-category in purple and 15-category in green.

Figure 5 shows the results when samples of 500 are simulated using 6 confounders (3 binary, 1 3-category, 1 4-category and 1 15-category). The inclusion of the 15-category variable has had little influence on the proportionate changes for the other factors in the unweighted model (Figure 2). When the factors are weighted the proportionate changes for the 4-category factor are increased in the expanded model as opposed to having decreased previously. Weighting of the prognostic factors has most effect on the extent to which the 15-category factor has a similar distribution for the 2 treatment groups.

## Discussion

In this paper we have simulated some common treatment trial scenarios and compared the results in terms of the distribution of discrepancies between treatment groups. Whilst only a selection of potential scenarios can be shown, we have illustrated how prior investigation helps quantify the sensitivity of minimisation to the choice of input parameters (randomisation weights, weighting of prognostic factors, number and type of factors). Assuming that numerically small differences between the groups are of little practical clinical importance, then we can make several useful statements regarding the selected scenarios:

• The number of factors to be taken into account can be increased quite substantially without severely affecting the overall balance.

• Weighting of the factors had most effect on the factors with many categories and was not highly detrimental to the factors with fewer categories (in keeping with the findings of Weir and Lees [13]).

• Including interaction terms in the minimisation did not greatly increase the overall discrepancies.

• Even weighting the randomisation by a small amount in favour of the preferred treatment had a large effect on the equality of the distribution of prognostic factors between treatments.

• Increasing the randomisation weights above 5 had little effect on the extent to which the treatment groups were similar.

Note that for a different number or type of minimisation factors the above statements may not hold. They are not meant to be universally true. These are statements about one particular hypothesised scenario to show how information relating to that scenario can be easily generated via simulations.

We believe that prior simulation according to expected sample size will be useful for clinicians embarking on a randomised controlled trial for which prognostic factors exist and should be equalised between treatment groups. Simulation will help quantify the effect of different input parameters on the expected discrepancies. It may assist in the choice of randomisation weighting utilised and the trade-off between minimizing for more criteria and/or increasing the categories of minimisation where prognostic factors are continuous. However, it should be noted that the decision of which variables to include in the minimisation process should be informed primarily by the clinical importance of variables and their impact on outcome. Vaughan Reed and Wickham [14] give further discussion to the choice of cut-points for continuous prognostic factors when performing minimisation. All minimisation variables should be adjusted for in the final analyses [1,9] and it is therefore important that only necessary variables are included to avoid over-parameterisation of the models. There is a trade-off between incorporating too many variables/unnecessarily increasing the number of categories used and allowing imbalance in important prognostic factors. The results of the simulation exercises can assist in the process but cannot be used as the sole, or even main, decision criteria for inclusion of a particular variable.

In theory, the process of minimisation is relatively simple to undertake [15] but in practice we feel that clinicians do not find it easy to keep running totals and perform weighted randomisations. Automation of the process (in addition to telephone allocation where available) reduces the propensity for conscious or unconscious interference with the allocation procedure. Given the importance of allocation concealment, this is an additional benefit of employing minimisation. Furthermore, simulated models are not necessarily straightforward to generate. Consequently, we have developed a software package (SiMin) for clinical usage which enables not only easy minimisation but also generates simulations that may be useful prior to the commencement of the study to help in determining which input parameters to use. It is important that the person performing the allocations is independent of the trial team. Having a quick and simple to use automated system makes it easier to enrol suitable individuals for this task.

At study commencement it should be possible to justify the number and type of minimisation variables, their weighting and the choice of randomisation weighting. Simulation enables estimation of the discrepancies anticipated and their probability. For example, suppose a treatment trial is estimated to require 40 patients to be allocated to the new or standard treatments to obtain a reasonable power to detect differences in outcome of clinical importance. Potential confounders are sex (male/female), age (under/over 18), whether the individual is an in- or out-patient, severity of disease (mild/moderate/severe) and ethnicity (4 categories). All categories of all minimisation variables are expected to be equally likely. (This latter criterion may not realistic but the density functions can be easily adjusted within the simulations.) If a randomisation weight of 2 (*p *= 0.67) is used then the proportionate change from expected of patients allocated to new and standard treatment is expected to be less than or equal to 0.35 for sex, age group and patient status (in- or out- patient), less than or equal to 0.45 for disease severity and less than or equal to 0.6 for ethnicity for 95% of random patient samples (figure 3). These are equivalent to absolute difference of 7, 6 and 6 patients respectively. A statement such as this could be incorporated into the protocol in a similar way to having a power calculation. i.e. the protocol could state, "Sex, age (under/over 18), residency status of patient (in/out), severity of disease (mild/moderate/severe) and ethnicity (4 categories) were included as factors in the minimisation. Factors were unweighted and a randomisation weighting of 2 was used for the allocations. It was estimated that the discrepancy between patients allocated to the 2 treatment groups would not exceed 7, 6 or 6 for the binary variables, disease state and ethnicity respectively with probability 0.95." Frequently, very little information is given on randomisation weights, weighting of variables or even the categories used in description of minimisation procedures.

Whilst it should be possible to justify the minimisation criteria, the precise details of the allocation process should not be widely divulged until after the trial has completed. The less information that is accessible, the less chance there is of recruitment being biased by knowledge of the likely allocation of future patients. We recommend that the details of and justification for the allocation process being employed are documented and given in the final trial reports. However, we also recommend that these details are not revealed to the research team during the trial apart from where this is essential.

The International Conference on Harmonisation E9 guidelines [16] discuss the importance of minimising bias in the design of trials, with 'bias' defined as "the systematic tendency of any factors associated with the design, conduct, analysis and interpretation of the results of clinical trials to make the estimate of a treatment effect deviate from its true value." These guidelines also state that "Good design should generally aim to achieve the same distribution of subjects to treatments within each centre and good management should maintain this design objective." The use of dynamic allocation of patients to treatments is one way to achieve these aims. In this paper we have investigated some of the issues that arise in the practical application of one allocation technique the use of which has risen sharply in recent years.

We have performed simulations using the most common scenario of 2 treatment groups. A similar process could be done where there are more treatment groups and this option has been incorporated into our software.

We have simulated datasets under different minimisation criteria to show how outcomes may vary as the input parameters are changed and suggest that this sort of approach should become standard practice. Previously it has been noted that simulations can be usefully employed prior to study commencement to determine the best allocation method to use [9,11,13]. These studies have used relatively complex models to compare not only minimisation parameters but also alternative approaches such as stratification. In some cases they have incorporated existing data [13]. Our results are in keeping with the findings of these more detailed studies. What we have aimed to show in this paper is how a relatively simple automatic algorithm, made available in package form, can be used to assist clinicians when they have decided to utilise minimisation and need to determine the optimal parameters. The package simplifies the practicalities of the process and hence may make this the preferred allocation method even when there are few prognostic factors to be taken into account and stratification is also a feasible option. It is important that researchers justify the choices they make with regards to the procedure for allocating patients. The arguments for this are not dissimilar to the argument for giving a power calculation or describing other details of the study protocol.

## Conclusion

The use of minimisation as a means of patient allocation is increasing. Decisions need to be made regarding the precise mode of implementation. Choice of input parameters may influence the extent to which the process is successful in ensuring equality of prognostic factors between treatment groups. We show how a simple automated package that we have developed locally can be used to allow researchers to investigate the effects of varying the input parameters prior to study commencement. The advent of the wide availability of computing technology makes minimisation a more realistic choice for many researchers. It is important that they utilise the technique most effectively. We have shown how simulations can be used prior to study commencement to ensure that the minimisation has a reasonable chance of providing comparable treatment groups.

## Competing interests

The author(s) declare that they have no competing interests.

## Authors' contributions

AW designed the study, performed the simulations and drafted the manuscript. HP provided computing support, verified the programs and created SiMin. SE, AP and EO provided the initial impetus for the study, tested the software and helped draft the manuscript. All authors read and approved the final manuscript.

## Pre-publication history

The pre-publication history for this paper can be accessed here:



## References

[B1] Taves DR (1974). Minimisation : A new method of assigning patients to treatment and control groups. Clinical Pharmacology and Therapeutics.

[B2] Pocock SJ, Simon R (1975). Sequential Treatment Assignment with Balancing for Prognostic Factors in the Controlled Clinical Trial. Biometrics.

[B3] Roberts C, Torgerson D (1998). Randomisation methods in controlled trials. BMJ.

[B4] Falk SJ, Girling DJ, White RJ, Hopwood P, Harvey A, Qian W, Stephens RJ (2002). Immediate versus delayed palliative thoracic radiotherapy in patients with unresectable locally advanced non-small cell lung cancer and minimal thoracic symptoms: randomised controlled trial. BMJ.

[B5] Pal DK, Das T, Chaudhury G, Johnson AL, Neville BGR (1998). Randomised controlled trial to assess acceptability of Phenobarbital for childhood epilepsy in rural India. The Lancet.

[B6] Hilton S, Doherty S, Kendrick T, Kerry S, Rink E, Steptoe A (1999). Promotion of healthy behaviour among adults at increased risk of coronary heart disease in general practice: methodology and baseline data from the Change of Heart study. Health Education Journal.

[B7] Altman DG, Bland JM (2005). Treatment allocation by minimisation. BMJ.

[B8] Buyse M, McEntegart (2004). Achieving balance in clinical trials: An unbalanced view from EU regulators. Applied Clinical Trials.

[B9] Scott NW, McPherson GC, Ramsay CR, Campbell MK (2002). The method of minimisation for allocation to clinical trials. Controlled Clinical Trials.

[B10] Senn S (2004). Controverises concerning randomization and additivity in clinical trials. Statistics in Medicine.

[B11] Atkinson AC (2002). The comparison of designs for sequential clinical trials with covariate information. JRSS A.

[B12] Bland JM, Altman DG (1989). Measuring agreement in method comparison studies. Statistical Methods in Medical Research.

[B13] Weir CJ, Lees KR (2003). Comparison of stratification and adaptive methods for treatment allocation in an acute stroke clinical trial. Statist Med.

[B14] Vaughan Reed J, Wickham EA (1988). Practical experience of minimisation in clinical trials. Pharmaceutical Medicine.

[B15] Freedman LS, White SJ (1976). On the use of Pocock and Simon's method for balancing treatment numbers over prognostic factors in the controlled clinical trial. Biometrics.

[B16] (1998). The International Conference on Harmonisation of Technical Requirements for Registration of Pharmaceuticals for Human Use  E9 Guidelines : Statistical Principles for Clinical Trials. http://www.ich.org.

